# Identification of IL-6 Signalling Components as Predictors of Severity and Outcome in COVID-19

**DOI:** 10.3389/fimmu.2022.891456

**Published:** 2022-05-13

**Authors:** María Ángeles Rodríguez-Hernández, David Carneros, María Núñez-Núñez, Ramón Coca, Rosario Baena, Gema M. López-Ruiz, María Elena Cano-Serrano, Alberto Martínez-Tellería, Ana Fuentes-López, Juan Manuel Praena-Fernandez, Christoph Garbers, José Hernández-Quero, Federico García, Stefan Rose-John, Matilde Bustos

**Affiliations:** ^1^ Area of Liver, Digestive and Inflammatory Diseases, Institute of Biomedicine of Seville (IBIS), Spanish National Research Council (CSIC) - University of Seville (US) - Virgen del Rocio University Hospital (HUVR), Seville, Spain; ^2^ Department of Pharmacy, San Cecilio University Hospital, Granada, Spain; ^3^ Infectious Disease Unit, San Cecilio University Hospital, Granada, Spain; ^4^ Biosanitary Research Institute of Granada (ibs.Granada), Granada, Spain; ^5^ Department of Clinical Analysis, Virgen de las Nieves University Hospital, Granada, Spain; ^6^ Department of Pharmaceutical Technology and Chemistry, University of Navarra, Pamplona, Spain; ^7^ Department of Anaesthesiology, San Cecilio University Hospital, Granada, Spain; ^8^ Department of Anaesthesiology, Virgen de las Nieves University Hospital, Granada, Spain; ^9^ Department of Microbiology, San Cecilio University Hospital, Granada, Spain; ^10^ Department of Statistics, Science Faculty, University of Granada, Granada, Spain; ^11^ Department of Pathology, Medical Faculty, Otto-von-Guericke-University Magdeburg, Magdeburg, Germany; ^12^ Centro de Investigación Biomédica en Red de Enfermedades Infecciosas (CIBERINFEC), ISCIII, Madrid, Spain; ^13^ Institute of Biochemistry, Kiel University, Kiel, Germany

**Keywords:** COVID-19, IL-6, soluble receptors, soluble IL-6 receptor (sIL-6R), soluble gp130 (sgp130), IL-6 trans-signalling

## Abstract

IL-6 is one of the major mediators of the hyper-inflammatory responses with complex biological functions as it can signal *via* different modes of action. IL-6 by classical signalling has anti-inflammatory and antibacterial activities, while trans-signalling mediates pro-inflammatory effects. The net biological effect of IL-6 is established by multiple factors beyond its absolute concentration. Here, we assess the relationship between IL-6 signalling variables [IL-6, soluble IL-6R (sIL-6R) and soluble gp130 (sgp130)] and outcomes in a cohort of 366 COVID-19 patients. The potential trans-signalling was evaluated by a ratio between the pro-inflammatory binary IL-6:sIL-6R complex and the inactive ternary IL-6:sIL-6R:sgp130 complex (binary/ternary complex) and the fold molar excess of sgp130 over sIL-6R (FME). Our data provide new evidence that high levels of IL-6, sIL-6R, sgp130, binary/ternary complex ratio, and low FME are independent predictors of COVID-19 severity in survivor patients (without death), and the combination of IL-6 + sIL-6R + sgp130 exhibited the most robust classification capacity. Conversely, in a subgroup of patients with a very poor prognosis, we found that high levels of IL-6 and low levels of sIL-6R, sgp130, and binary/ternary complex ratio were predictors of death. In this context, the highest predictive capacity corresponded to the combined analysis of IL-6 + FME + lymphopenia + creatinine. Herein, we present IL-6 signalling variables as a helpful tool for the early identification and stratification of patients with clear implications for treatment and clinical decision-making.

## Introduction

Coronavirus disease 2019 (COVID-19) is a complex disease caused by severe acute respiratory syndrome coronavirus-2 (SARS-CoV-2) infection (1). Clinical manifestations of COVID-19 range from asymptomatic to acute respiratory distress syndrome (ARDS). It is unknown how to predict rapid respiratory failure and severity in COVID-19 patients.

One of the major pathological characteristics of SARS-CoV-2 infection is the excessive responsive secretion of inflammatory cytokines, followed by the attack of the immune system in various tissues, which causes ARDS (2). In severe COVID-19 patients after the inflammatory outburst, the immune system may be programmed to an immune suppression condition, which leads to death (3). It was observed that patients with severe disease had high levels of inflammatory markers, including C-reactive protein (CRP), ferritin, D-dimer, high neutrophil-to-lymphocyte ratio, and increased levels of cytokines (4); thus, comparing COVID-19 disease to CAR-T (chimeric antigen receptor-modified T cells) cytokine release storm (CRS) (5). The proposed central role of a cytokine storm in COVID-19 disease escalation prompts the question of whether interleukin (IL)-6 normalization strategies during the maladapted inflammatory response could be useful. Indeed, tocilizumab, an anti–IL-6 receptor monoclonal antibody, was used in COVID-19. However, despite an effect on mortality being initially suggested by several retrospective studies (6–9), prospective clinical trial data have been less convincing (10–13). These facts clearly show an urgent need to identify biomarkers in order to help guide rational targeted immunomodulatory therapeutic strategies.

IL-6 is produced in response to infection and tissue damage and exhibits a complex biology as it can signal *via* different modes of action (14). In classic signalling, IL-6 binds to the membrane-bound IL-6R, which is mainly expressed on hepatocytes and immune cells, and induces homodimerization of glycoprotein 130 (gp130). In the trans-signalling mode, IL-6 uses the soluble form of the IL-6R (sIL-6R). The IL-6:sIL-6R complex also induces gp130 homodimerization. IL-6 trans-signalling enables cells lacking the membrane-bound IL-6R to be stimulated by IL-6. This mode of signalling is considered to cause proinflammatory properties and its selective inhibition holds the promise to cause less unwanted side effects compared to global IL-6 blockade (15). Notably, soluble variants of gp130 (sgp130), can inactivate the IL-6:sIL-6R binary complex and disrupt IL-6 trans-signalling. Thus, the ternary IL-6:sIL-6R:sgp130 complex is unable to bind and activate cellular gp130, and high levels of sIL-6R and sgp130 constitute a sort of buffer for IL-6 (16). Several studies have shown disturbances in the natural IL-6:sIL-6R:sgp130 buffer in pathologies such as infections or CRS (17), type 2 diabetes (18), cardiovascular diseases (19), and in chronic inflammation such as experimental atherosclerosis (20). Active IL-6 trans-signalling influences T-cell recruitment, activation, and apoptosis. It can boost differentiation of Th17 cells and at the same time suppress the development of regulatory T cells (21). IL-6 trans-signalling is the major route of IL-6 signalling to microvascular endothelial cells (EC) (22), leading to EC activation, dysfunction, and deregulated inflammatory cell infiltration (23). It has been implicated in the endotheliopathy in COVID-19 (24, 25), with a central role in the pathogenesis of ARDS and multi-organ failure (23).

Our present study was designed to investigate the ability of the different components in serum of IL-6 signalling including IL-6, sIL-6R, and sgp130 to discriminate severity and to predict disease course and outcome in COVID-19 patients. We also wanted to study the different approaches used to investigate potential trans-signalling, analysing the IL6:sIL-6R binary and the IL-6:sIL-6R:sgp130 ternary complexes, and the fold molar excess of sgp130 over sIL-6R (FME) in serum. Our results suggest that the profiling of those proteins could be used to stratify patients, guide resource allocation, and interventional studies. In addition, our results could help to understand the IL-6:sIL-6R axis in SARS-CoV-2 infection with important implications in the treatment of this disease.

## Methods

### Patients and Data Collection

In this study we used SARS-CoV-2-positive symptomatic adult patients admitted to two different hospitals in Spain (San Cecilio University Hospital, Granada, n = 308 and Virgen de las Nieves University Hospital, Granada, n = 58) between 03/13/2020 and 03/07/2021. Diagnosis of SARS-CoV-2 infection was confirmed by real-time reverse transcription-polymerase chain reaction method from the nasopharyngeal swab (according to World Health Organization interim guidance). Data on demographic characteristic, clinical (including both previous comorbidities as well as COVID-19 related information), and laboratory data were obtained from electronic medical records (see **Supplementary Tables S1, S2**, respectively). We evaluated the hospitalization time and evolution in terms of hospital discharge, internal care unit (ICU) admission, and/or death. Blood samples were collected typically upon admission to the hospital (median, 1 day; IQR 1-4), and laboratory results were recorded on the day of the blood extraction. In concordance with other studies (26–28), our cohort was classified as i) moderate (hospital ward, respiratory symptoms, and/or radiological evidence of pneumonia without requirement of mechanical ventilation), ii) ICU (radiological evidence of pneumonia and/or requirement of mechanical ventilation and/or intensive care unit (severe survivors), and iii) exitus (severe non-survivors).

### Ethical Commitment

The study was conducted according to the principles of the Declaration of Helsinki (7^th^ revision) with the local ethics committee (Biomedical Research Ethics Committee of Andalucía code: COVIDIL6; Andalucía Biobank code: S2000228). The study had no risk or negative consequence on those who participated in the study. Medical record numbers were used for data collection without using personal identifiers. An anonymous identification code was assigned to each medical record number to assure data protection policy.

### IL-6 and Soluble Receptors Analysis

Patient’s serum was used to determine IL-6, sIL-6R, and sgp130 levels using human IL-6 ELISA Kit (Immunotools, Friesoythe, Germany), DuoSet^®^ ELISA Human IL-6Rα (R&D Systems, Minneapolis, MN, USA) and DuoSet^®^ ELISA human gp130 (R&D Systems) respectively. Analyses were performed according to manufacturer’s instructions.

### Derivation of Molar Concentration of Different Components of IL-6 Signalling and the Binary and Ternary Complexes

We calculated IL-6, sIL-6R, and sgp130 molar (M) concentration from each patient according with their respective molecular weights (IL-6 = 23.7 kDa, sIL-6R = 50 kDa, and sgp130 = 100 kDa). Regarding sgp130, it has been described different natural soluble isoforms of sgp130 with different molecular masses (29, 30) and we applied use the one used by Müller- Newen (31). Next, we estimated the M concentration of both binary (IL-6:sIL-6R) and ternary (IL-6:sIL-6R:sgp130) complexes, according with the formulas described by Ziegler et al. (19) (originally presented by Müller-Newen et al.) (31).


(1) $$\left[\text{IL}-6:\text{IL}-6\text{R}\right]\;=\;0.5\left[\text{sIL}-6\text{R}\right]\text{i}+0.5\left[\text{IL}-6\right]\text{i}+0.5{\text{K}}_{\text{D}1}–0.5\left({\left[\text{sIL}-6\text{R}\right]}^{2}+\left[\text{IL}-6\right]{\text{i}}^{2}+2\left[\text{IL}-6\right]{\text{iK}}_{\text{D}1}+{\text{K}}_{\text{D}1}^{2}\right)0.5$$

(2) $$\left[\text{IL}-6:\text{sIL}-6\text{R}:\text{sgp}130\right]=0.5\left[\text{sgp}130\right]\text{i}+0.5\left[\text{IL}-6/\text{sIL}-6\text{R}\right]\text{i}+0.5{\text{K}}_{\text{D}2}–0.5\left(\left[\text{sgp}130\right]{\text{i}}^{2}+\left[\text{IL}-6:\text{sIL}-6\text{R}\right]{\text{i}}^{2}\;+\;2\left[\text{IL}-6:\text{sIL}-6\text{R}\right]{\text{iK}}_{\text{D}2}\;+{\text{ K}}_{\text{D}2}^{2}\right)0.5$$

[IL-6]i, [sIL-6R]i and [sgp130]i were substituted with their respective M concentration. K_D1_ and K_D2_, which represent the dissociation constants for the binary and ternary complex, were 0.5 nM and 0.05 nM, respectively (31). The binary/ternary complex ratio (hereinafter B/T complex ratio) was calculated by dividing nM concentration of binary complex by nM concentration of ternary complex.

Moreover, we calculated the molar excess of sgp130 over IL-6R, considering that sgp130 exists predominantly as a dimer: [sIL-6R nM]: [2x sgp130 nM] ratio (18). The results are presented as a fold of the molar excess sgp130 over sIL-6R (hereinafter FME).

### Statistical Analysis

Patient characteristics were summarized using the standard descriptive statistical methods: median (interquartile range) for continuous variables and number (percentage) for categorical variables. The normal distribution of outcome variables was analysed using the Kolmogorov–Smirnov test. For continuous variables, the differences were computed using Mann-Whitney U test, Kruskal–Wallis test and Spearman´s rank correlation. Categoric variables were compared by using a Chi-square test or Fisher exact test when appropriate. Box plots were represented in Tukey style. Each variable of interest was evaluated as a potential biomarker by using area under receiver operating characteristic (AUROC) curves. DeLong’s test evaluated statistically significant differences among ROC curves. The Youden’s index was used for cut-off selection, and cytokines were then categorized at their corresponding cut-off levels. Sensitivity, specificity, negative predictive value (NPV), and positive predictive value (PPV) were calculated for each individual variable and model cut-off. The univariate logistic regression analyses assessed the association of the cytokines with patient outcome. Multivariable logistic regression analyses were performed considering those variables statistically significant (5% level) in the univariate analyses, avoiding combinations of variables that would lead to collinearity. Kaplan–Meier plots were conducted to assess the differences survival probabilities regarding levels of cytokines (above *vs* below cut-offs) across the follow-up timeframe, which was calculated from the date of hospital admission to date of discharge, end of follow-up period, or death (restricted 30 days). Log-rank test or Breslow (when appropriate) was performed to screen the significant candidate indicators. The COX regression hazards model estimated the hazard of death. Omnibus tests assessed the models’ goodness of fit. Throughout the analysis, only patients with available data were compared. GraphPad Prism 8.4.2, IBM SPSS Statistics 22 software and R studio i386 4.1.2 (libraries pROC, survival, survminer, survMisc, and ggplot2) were used. Internal validation of the proposed models was performed by bootstrapping with 1000 replications.

#### Construction of the Scoring Models

Different models were built to test the predictive/prognostic capacity of the association of different variables. In these models, each variable was weighted by its risk factor obtained in the multivariate logistic regression analysis. The overall risk score for each model was obtained by summing the weights thereby obtained from all coefficients (32). Afterwards, the final risk score model was assessed using the AUROC curve and the Omnibus goodness of fit test. Forest plots of OR (odds ratio) and HR (hazard ratio) summarized the risk of severity/mortality for increasing values of the score.

## Results

### Cohort Characteristics and IL-6 Signalling Variables Ranges

A total of 366 SARS-CoV-2 positive symptomatic patients were included in the study. To assess whether IL-6 signalling was important to determine the severity of the disease, patients were classified into moderate (n = 257, 70.20%), severe survivors (ICU; n = 40, 10.9%), and severe non-survivors (exitus; n = 69, 18.9%). Clinical and demographic characteristics of the patients and biochemical data at hospital admission are reported in **Supplementary Tables S1, S2**. In brief, the median age of non-survivors was significantly older (78 years old) than the severe group (62.5 years old), with a higher proportion of male subjects, 83.3% *vs* 60%. The most common comorbidities in the three groups were hypertension (44.7% in moderate, 32.5% in severe survivors and 71% in non-survivors), diabetes mellitus (23.7% in moderate, 25% in severe survivors, and 36% in non-survivors), and dyslipidaemia (23.7% in moderate, 25% in severe survivors, and 25% in non-survivors). Notably, hypertension, renal, and heart disease were significantly present in higher proportion of participants in the exitus group *vs* severe and moderate patients. Chronic pulmonary disease was significantly present in severe subjects (survivors and exitus) compared to moderates. Symptoms such as fever (73.9%), dyspnoea (44.7%), anosmia (16.43%), and dysgeusia (20.43%) were characteristic of the moderate group (**Supplementary Table S1**).

**Figure 1:**
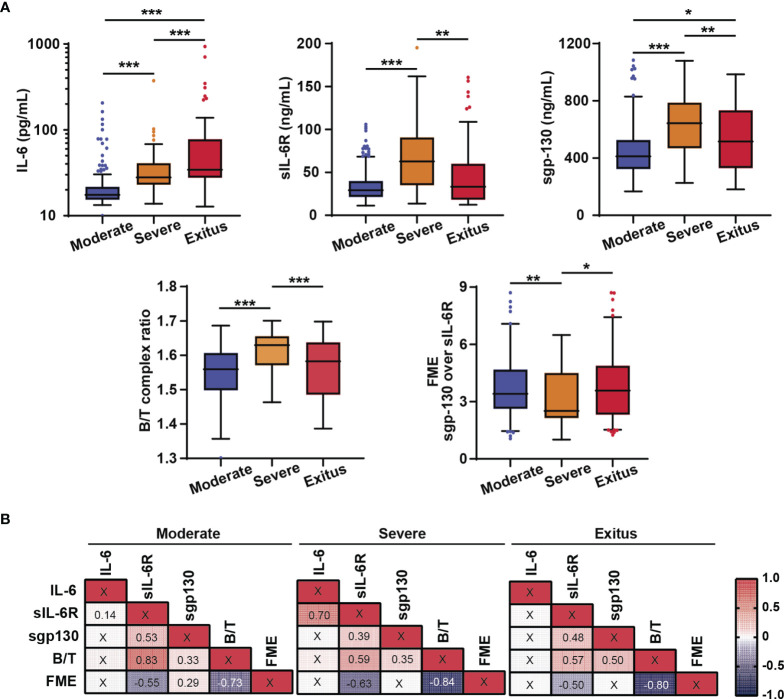
Analysis of IL-6 signalling biomarkers. **(A)** Serum levels of IL-6, sIL-6R and sgp130, and Binary/Ternary complex ratio (B/T) and fold molar excess of sgp130 over sIL-6R (FME) in moderate (n = 257), severe survivors (n = 40) and severe non-survivors (exitus) (n = 69) hospitalized COVID-19 patients. Box plots showed median with upper and lower quartiles. The whiskers show Tukey confidence intervals. **(B)** Correlation matrix of IL-6 signalling variables assessed using Spearman’s Rho analysis. Statistically significant results are shown with the corresponding correlation coefficients. Binary/Ternary complex ratio (B/T); Fold molar excess of sgp130 over sIL-6R (FME). *p < 0.05; **p < 0.01; ***p < 0.001; “X” no statistical significance.

In order to elucidate if IL-6 signalling was helpful to discriminate severe COVID-19, we analysed serum levels of IL-6, sIL-6R, and sgp130. Moreover, both B/T complex ratio (19, 33) and the combination of IL-6 levels and FME (16) have been included in the analysis as indicators of IL-6 trans-signalling. Serum levels of IL-6, sIL-6R, sgp130, and B/T complex ratio were significantly increased in severe survivors compared to moderates: IL-6 (moderate 17.50 pg/mL *vs* severe 27.60 pg/mL), sIL-6R (moderate 29.15 ng/mL *vs* severe 62.78 ng/mL), sgp130 (moderate 411.71 ng/mL *vs* severe 644.10 ng/mL), and B/T complex ratio (moderate 1.56 *vs* severe 1.64). Conversely, FME decreased significantly in severe survivors (moderate 3.41 *vs* severe 2.45). Surprisingly, in the exitus group only the levels of IL-6 were significantly increased compared to severe survivors (34.42 pg/mL *vs* 27.60 pg/mL, respectively), while the levels of sIL-6R (33.31 ng/mL *vs* 62.78 ng/mL, respectively), sgp130 (515.42 ng/mL *vs* 644.1 ng/mL, respectively), and B/T complex ratio (1.57 *vs* 1.64, respectively) were significantly decreased. FME showed an upward trend (**Figure 1A**). On the other hand, the levels of sIL-6R and sgp130 positively correlated in all groups of patients (**Figure 1B**). Notably, most of the subjects in severe and exitus groups had sIL-6R levels matched with sgp130, but increased IL-6 did not imply high values in neither of sIL-6R nor sgp130 (**Supplementary Figure S1**). Furthermore, in severe survivors but not in exitus, levels of IL-6 positively correlated with sIL-6R (**Figure 1B**).

Correlation analyses between the levels of IL-6 signalling-related variables with biochemical and haematological parameters showed a positive correlation of IL-6 with CRP values in all groups of patients and with neutrophils/lymphocytes ratio, platelets/lymphocytes ratio, and systemic immune-inflammation index only in moderates. On the other hand, levels of sIL-6R and sgp130 as well as B/T complex ratio, but not IL-6, positively correlated with ferritin (a marker of inflammation and severity) only in severe survivors. Lactate dehydrogenase, neutrophils/lymphocyte ratio, glucose, and potassium levels positively correlated with sIL-6R and sgp130 in the exitus group (**Supplementary Figure S2**). Altogether, the data evidence that soluble receptors, sIL-6R and sgp130, rather than IL-6, could characterize COVID-19 severity.

### The Value of IL-6 Signalling Components as Predictors of Disease Progression

**Figure 2:**
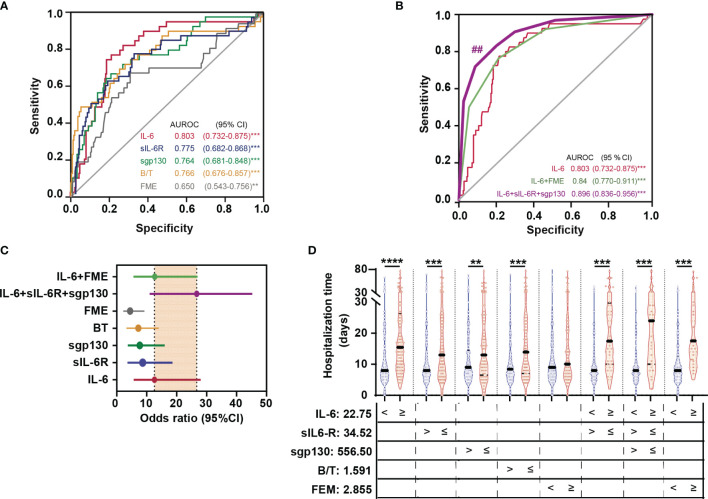
IL-6 signalling biomarkers as predictors of severity without dead. AUROC curves (95% CI) of IL-6 signalling variables **(A)**, and scores combining IL-6 signalling variables **(B)**. Statistically significant differences among ROC curves were evaluated using DeLong’s test ^##^ *p* < 0.01 *vs* IL-6 ROC curve. **(C)** Forest plots of OR summarizing the risk of severity for each variable or proposed score. **(D)** Violin plots indicating length of hospital stay (days) in patients according with levels of IL-6, sIL-6R, sgp130, Binary/Ternary complex ratio (B/T), and fold molar excess of sgp130 over sIL-6R (FME) above and below cut-off values. Each dot represents a sample; solid and dotted lines show the median and 95% CI, respectively. **p < 0.01; ***p < 0.001.

Analysis of the AUROC curve was used to assess the efficacy of IL-6 signalling-related variables as potential discriminators of COVID-19 severity (focused on severe patients who survived the disease). Results showed significant predictive value for all of them: IL-6 cut-off 22.75 pg/mL, AUROC 0.80 (0.73 - 0.88), 77.5% sensitivity and 78.5% specificity; sIL-6R cut-off: 34.52 ng/mL, AUROC 0.78 (0.68 - 0.89), 79.5% sensitivity and 67.9% specificity; sgp130 cut-off 556.50 ng/mL, AUROC 0.76 (0.68 – 0.85), 66.0% sensitivity and 79.4% specificity; B/T complex ratio cut-off 1.59, AUROC 0.77 (0.68 - 0.86), 74.4% sensitivity and 69.1% specificity; FME cut-off 2.86, AUROC 0.65 (0.54 - 0.76), 65.8% sensitivity and 69.9% specificity (**Figure 2A** and **Table 1**). Univariate logistic regression analyses showed that all IL-6 signalling components acted as significant individual predictors of severity. Consequently, patients with levels of IL-6 signalling variables above cut-offs presented a significant increased risk of developing severe COVID-19 (IL-6: OR 12.59 (5.66 - 28.0); sIL-6R: OR 8.19 (3.60 - 18.61); sgp130: OR 7.69 (3.69 - 16.01)). In the same line, levels of B/T complex ratio above cut-off and FME below cut-off had increased risk of severity (B/T: OR 7.21 (3.25-15.97); FME: OR 4.47 (2.17-9.22)) (**Table 1**). Finally, IL-6, sIL-6R, and sgp130, as well as the IL-6 and FME, were included in multivariate models, remaining as significant co-predictors of severity (**Table 1**). The same conclusion was found after adjustment by gender and age (**Supplementary Table S3A**).

**Table 1:** Univariate and multivariate logistic regression analyses of IL-6 signalling components as predictors of COVID-19 severity (without death).

	Risk factor cut-off characterization					Cut-off univariate logistic		Cut-off multivariate logistic		Cut-off multivariate logistic	
Variable	Cut-off	Sensitivity %	Specificity %	PPV %	NPV %	OR	95% CI	OR	95% CI	OR	95% CI
**IL-6** (pg/mL)	≥ 22.75	77.5	78.5	36.5	95.7	12.59	(5.66-28.00)***	9.87	(4.15-23.43)***	13.37	(5.69-31.09)***
**sIL-6R** (ng/mL)	≥ 34.52	79.5	67.9	27.9	95.5	8.19	(3.60-18.61)***	4.36	(1.71-11.17)***	–	–
**sgp130** (ng/mL)	≥ 556.50	66.0	79.4	33.8	93.8	7.69	(3.69-16.01)***	3.86	(1.63-9.18)***	–	–
**B/T**	≥ 1.591	74.4	69.1	29.8	95.0	7.21	(3.25-15.97)***	–	–	–	–
**FME**	≤ 2.855	65.8	69.9	25.3	93.0	4.47	(2.17-9.22)***	–	–	5.09	(2.25-11.52)***

NPV, Negative predictive value; PPV, Positive predictive value; B/T, Binary/ternary complex ratio; FME, Fold molar excess of sgp130 over sIL-6R; OR, Odds ratio. ***p < 0.001.

To better understand the predictive roles of the three components of IL-6 signalling as well as the markers of potential IL-6 trans-signalling (B/T complex ratio and FME) in the severity of the disease (without death), we converted the ORs obtained in the multivariate regression analysis into integer single risk scores and constructed a total risk score by summing the component variables (32, 34) (**Table 2**). Statistical analysis predicted excellent classification performance for severity in survivors using the proposed scores. The maximum AUROC was obtained for the combination of IL-6 + sIL-6R + sgp130 [0.90 (0.84 - 0.96)], with significant differences *vs* IL-6 AUROC curve [0.80 (0.73 - 0.88)]. In addition, the model based on IL-6 + sIL-6R + sgp130 increased values of sensitivity, specificity, accuracy, and OR (77.8%, 91.29%, 89.01%, and 26.72, respectively), compared to IL-6 (77.5%, 78.52%, 79.38%, and 12.59, respectively) (**Figures 2B, C** and **Table 2**). The findings clearly show the robustness of the predictive model based on the three mentioned variables, being superior to the exclusive use of IL-6. Supporting these data, it was observed that subjects with initial levels of IL-6, sIL-6R, sgp130, and B/T complex ratio above stablished cut-offs and FME below cut-off required prolonged hospitalization. Notably, patients who needed a longer length of hospital stay were those with increased levels of IL-6 + sIL-6R + sgp130 (median 24 days *vs* 15.5 days in patients with exclusively elevated IL-6) (**Figure 2D**).

**Table 2:** Univariate logistic regression analyses of IL-6 signalling components as predictors of severity based on proposed scores.

Variable	Score	Risk factor cut-off characterization						Cut-off univariate logistic	
		Variable	Score	Cut-off	Sensitivity %	Specificity %	PPV %	NPV %	Accuracy %	OR	95% CI
**IL-6**	–	≥ 22.75	77.5	78.5	36.5	95.7	79.38	12.59	(5.66-28.00)***
**IL-6+sIL-6R+sgp130**	0-18	≥ 12	77.88	91.29	52.27	96.07	89.01	26.72	(10.98-45.27)***
**IL-6+FME**	0-18	≥ 9	76.60	78,82	36,3	95,5	78,49	12.69	(5.71 - 29.11)***

NPV, Negative predictive value; PPV, Positive predictive value; B/T, Binary/ternary complex ratio; FME, Fold molar excess of sgp130 over sIL-6R; OR, Odds ratio. ***p < 0.001.

**Figure 3:**
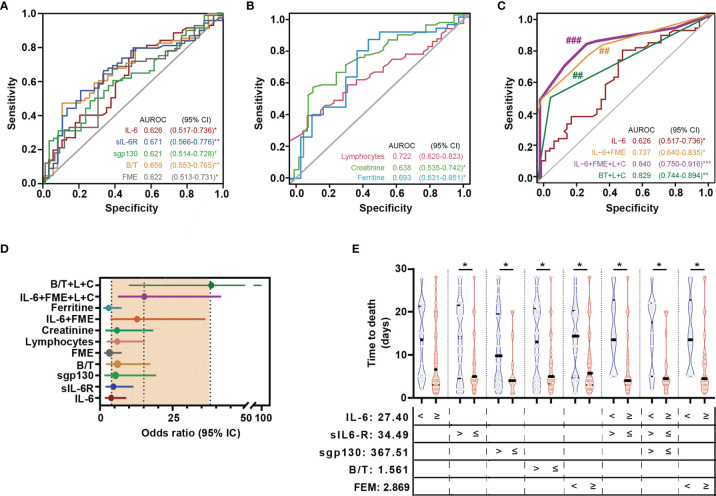
IL-6 signalling biomarkers as predictors of mortality. AUROC curves (95% CI) of IL-6 signalling variables and biochemical data **(A)** and composed scores **(B)**. Statistically significant differences among ROC curves were evaluated using DeLong’s test. ^##^ *p* < 0.01 *vs* IL-6 ROC curve. **(C)** Forest plots of OR summarizing the risk of death for each variable or proposed score. **(D)** Violin plots indicating time to death according with levels of IL-6, sIL-6R, sgp130, Binary/Ternary complex ratio (B/T), and fold molar excess of sgp130 over sIL-6R (FME) above and below cut-off values. Each dot represents a sample; solid and dotted lines show the median and 95% CI, respectively. (L) lymphopenia and (C) creatinine. ***p < 0.001, *p < 0.05; **p < 0.01; ^###^p < 0.001 vs IL-6 ROC curve.

In order to get further insight into the value of IL-6 signalling variables in the prediction of death in COVID-19, new analyses were performed focused on severe patients (survivors and non-survivors). Notably, the risk of death corresponded to patients with values of sIL-6R, sgp130, and B/T complex ratio below stablished cut-offs (34.49 ng/mL, 367.51 ng/mL, and 1.56, respectively) and IL-6 and FME above cut-offs (27.4 pg/mL and 2.87, respectively). As reflected in **Figure 3A** and **Table 3A**, all the variables were significant individual predictors of death in univariate logistic regression analyses: IL-6 AUROC 0.63 (0.52-0.74), 79.4% sensitivity and 50.04% specificity; sIL-6R AUROC 0.67 (0.57-0.78), 54.4% sensitivity and 79.5% specificity; sgp130 AUROC 0.62 (0.51-0.73), 31% sensitivity and 92.3% specificity; B/T complex ratio AUROC 0.66 (0.55-0.77), 47.1% sensitivity and 84.7% specificity; FME AUROC 0.62 (0.51-0.73), 64.7% sensitivity and 65.2% specificity. Patients who presented the highest risk of death corresponded to B/T complex ratio values below cut-off [OR: 6.05 (2.11-17.33)]. Moreover as reflected in **Table 3B**, both B/T complex ratio as well as the covariables IL-6 and FME remained significant predictors of death in the multivariate model even adjusted by ferritin, creatinine, and lymphocytes (biochemical data with significant differences between severe survivor and exitus groups, **Supplementary Table S2**). The same conclusions were found after corrections by gender and age (**Supplementary Table S3B**). Based on the ORs obtained in the multivariate model, different risk scores were constructed (**Table 4**). The maximum predictive value corresponded to the combination of IL-6 + FME + lymphopenia + creatinine [AUROC 0.83 (0.75 -0.92), 86.27% sensitivity, 63.16% specificity, 76.4% accuracy, and OR 10.78 (3.83 - 30.32)] (**Figures 3B, C** and **Table 4**). All patients with levels of IL-6 and B/T complex ratio above cut-off and sIL-6R, sgp130 and FME below cut-offs died in the first week of hospitalization. The lowest survival time corresponded to patients with increased IL-6 and decreased sIL-6R and sgp130 levels (**Figure 3D**). Altogether, the data indicate that IL-6 signalling markers as well as the indicators of IL-6 trans-signalling are efficient predictors of COVID-19 disease progression in terms of severity and mortality.

**Table 3A:** Univariate logistic regression analyses of IL-6 signalling biomarkers as predictors of COVID-19 mortality.

Variable	Risk factor cut-off characterization					Cut-off univariate logistic	
	Cut-off	Sensitivity %	Specificity %	PPV %	NPV %	O.R.	95% CI
**IL-6** (pg/mL)	≥ 27.40	79.40	50.04	73.00	58.81	3.86	(1.64-9.06)*
**sIL-6R** (ng/mL)	≤ 34.49	54.44	79.49	82.27	50.04	4.63	(1.86-11.51)***
**sgp130** (ng/mL)	≤ 367.51	30.96	92.30	87.56	43.46	5.36	(1.48-19.39)***
**B/T**	≤ 1.561	47.11	84.67	88.93	49.37	6.05	(2.11-17.33)**
**FME**	≥ 2.869	64.74	65.18	75.93	51.05	3.27	(1.44-7.45)**
**Lymphocytes** (x 10^3/µL)	≤ 0.565	39.39	90.00	62.26	–	5.96	(2.30-15.41)***
**Creatinine** (U/L)	≥ 1.15	57.35	81.58	84.78	51.67	5.85	(1.86-18.38)**
**Troponine** (ng/mL)	≥ 12.05	84.44	56.25	84.44	19.15	6,98	(1.95-24.98)**
**Ferritine** (ng/mL)	≥ 992.75	76.59	45.95	64.29	32.08	2.78	(1.09-7.09)**

NPV, Negative predictive value; PPV, Positive predictive value; B/T, Binary/ternary complex ratio; FME, Fold molar excess of sgp130 over sIL-6R; O.R., Odds ratio. *p < 0.05, **p < 0.01, ***p < 0.001.

**Table 3B:** Multivariate logistic regression analyses of IL-6 signalling biomarkers as predictors of COVID-19 mortality.

Variable	O.R.	95% CI	O.R.	95% CI	O.R.	95% CI	O.R.	95% CI	O.R.	95% CI
**IL-6** ≥ 27.40 pg/mL	2.42	(0.96-6.01)	3.69	(1.33-10.28)*	4.08	(1.62-10.3)**	4.05	(1.30-12.61)*	–	–
**sIL-6R** ≤ 34.49 ng/mL	3.91	(1.39-11)**	–	–	–	–	–	–	–	–
**sgp130** ≤ 367.51 ng/mL	2.02	(0.48-8.43)	–	–	–	–	–	–	–	–
**B/T** ≤ 1.561	–	–	–	–	–	–	–	–	5.85	(1.82-18.81)**
**FME** ≥ 2.869	–	–	–	–	3.67	(1.50-9)**	4.39	(1.51-12.71)**	–	–
**Lymphocytes** ≤ 0.565 x 10^3/µL	–	–	5.02	(2.43-19.86)***			6.71	(1.60-28.10)**	4.87	(1.37-17.29)**
**Creatinine** ≥ 1.15 U/L	–	–	6.95	(1.49-18.17)*			6.78	(2.17-21.11)**	7.38	(2.49-21.92)***

NPV, Negative predictive value; PPV, Positive predictive value; B/T, Binary/ternary complex ratio; FME, Fold molar excess of sgp130 over sIL-6R; O.R., Odds ratio. *p < 0.05, **p < 0.01, ***p < 0.001.

**Table 4:** Univariate logistic regression analyses of IL-6 signalling components as predictors of COVID-19 mortality based on proposed scores.

Variable	Score	Risk factor cut-off characterization						Cut-off univariate logistic	
		Cut-off	Sensitivity %	Specificity %	PPV %	NPV %	Accuracy %	OR	95% CI
**IL-6**	–	27.40	72.97	58.82	79.41	50	68.52	3.86	(1.64-9.06)**
**IL-6+FME**	0-8	6	52.31	92.11	91.90	53.03	66.99	12.80	(3.57-45.82)***
**IL-6+Lymphopenia+Creatinine**	0-16	8	70.31	73.68	81.82	59.57	71.57	6.63	(2.70-13.30)***
**IL-6+FME+Lymphopenia+Creatinine**	0-22	8	85.25	72.22	83.88	74.29	80.41	15.02	(5.44-41.500)***
**B/T+Lymphopenia+Creatinine**	0-18	9	51.56	97.30	97.06	53.73	68.32	38.32	(4.95-166.67)***

NPV, Negative predictive value; PPV, Positive predictive value; B/T, Binary/ternary complex ratio; FME, Fold molar excess of sgp130 over sIL-6R; OR, Odds ratio. **p < 0.01; ***p < 0.001.

### The Value of IL-6 Signalling Components as Prognostic Indicators of Survival

**Figure 4:**
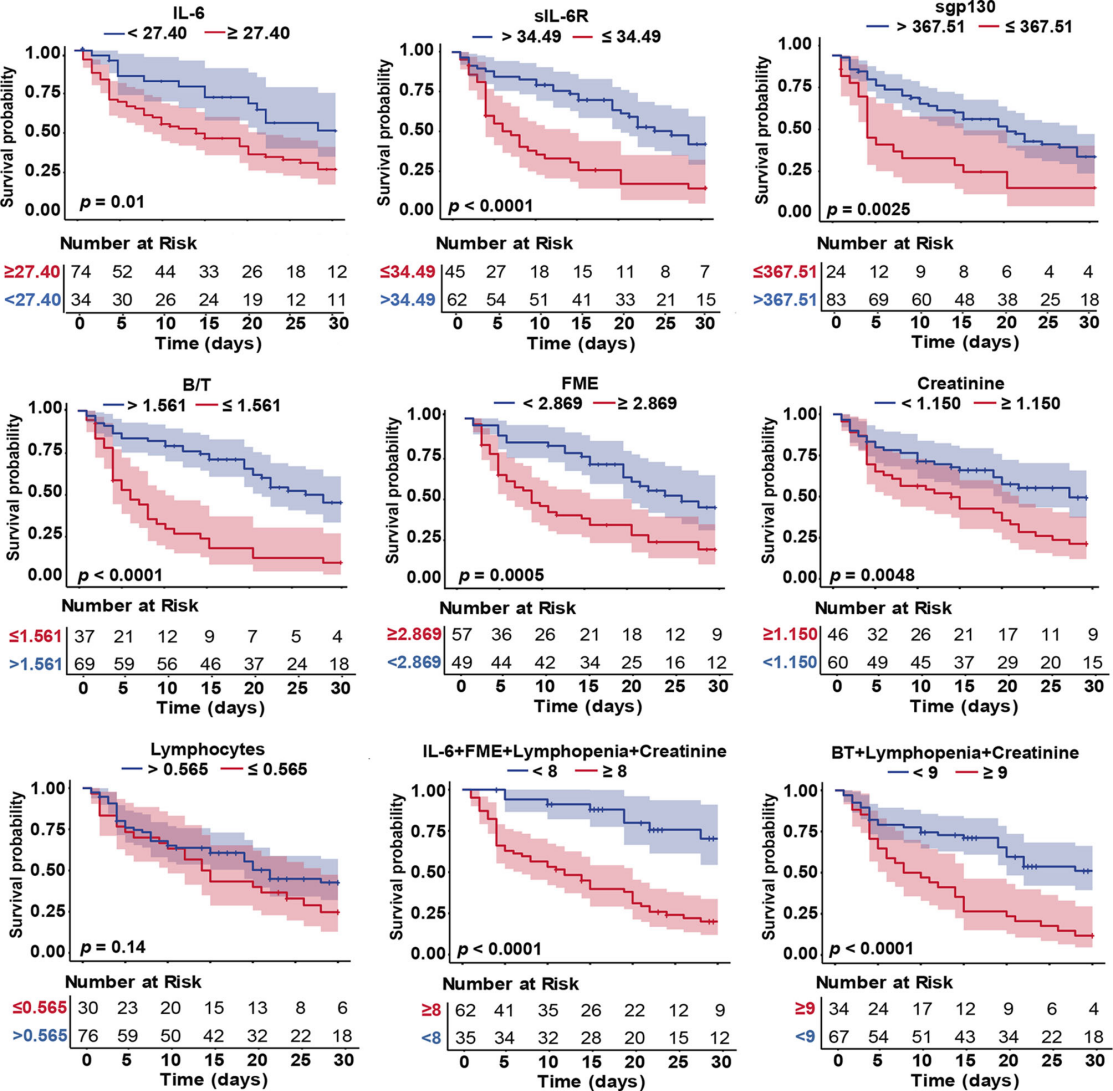
Kaplan-Meier curves of IL-6 signalling biomarkers, biochemical data and composite risk scores in COVID-19 severe patients. Parameters of Kaplan-Meier survival analysis: events (death); time to events (time from hospital admission to death) and end-point (30-day). Differences in time to death curves were compared with the log-rank/Breslow tests. Colour shades represent the 95% CI.

Kaplan-Meier curves were conducted to further evaluate the prognostic value of the variables involved in IL-6 signalling for severe group (survivors and exitus). Results showed that increased levels of IL-6 and FME, and decreased levels of sIL-6R, sgp130, and B/T complex ratio were significantly associated with decreased cumulative survival rate during the follow-up timeframe. All IL-6 signalling components served as independent predictors of death within 30 days. Moreover, when patients were categorized into high- and low- risk groups based on the proposed scores, Kaplan-Meier curves showed that most of the deceased cases corresponded with the high-risk group, whereas the alive cases were associated with the low-risk group, with a significant divergence in the overall survival outcomes (**Figure 4**). Subsequent COX regression analysis showed that models including B/T complex ratio as well as FME, both variables mirroring IL-6 trans-signalling, presented strong prognostic capacity even after adjustment for creatinine and lymphopenia (biochemical data with significant differences between severe survivors and severe non-survivors, **Supplementary Table S1**). Hazard of death for patients categorized in the high-risk according to IL-6 was 2.27 (1.23 - 4.18), while for combinations of IL-6 + FME + lymphopenia + creatinine and B/T complex ratio + lymphopenia + creatinine increased to 5.12 (2.51 – 10.41) and 3.02 (1.84 - 4.97), respectively (**Table 5**).

**Table 5:** COX regression analyses of IL-6 signalling components as predictors of COVID-19 mortality based on proposed scores.

	COX regression analysis	
Variable	HR	95% CI
**IL-6** ≥ 27.40 pg/mL	2.27	(1.23-4.18)**
**sIL-6R** ≤ 34.49 ng/mL	2.50	(1.517-4.14)***
**sgp130** ≤ 367.51 ng/mL	2.25	(1.31-3.87)**
**B/T** ≤ 1.561	3.10	(1.87-5.15)**
**FME** ≥ 2.869	2.32	(1.38-3.91)**
**IL-6+FME** ≥ 6	3.28	(2.00-5.37)***
**IL-6+Lymphopenia+Creatinine** ≥ 8	2.53	(1.48-4.33)**
**IL-6+FME+Lymphopenia+Creatinine** ≥ 8	5.12	(2.51-10.41)***
**B/T+Lymphopenia+Creatinine** ≥ 9	3.02	(1.84-4.97)***

B/T, Binary/ternary complex ratio; FME, Fold molar excess of sgp130 over sIL-6R; HR, Hazard ratio. **p < 0.01; ***p < 0.001.

Finally, a representative model of IL-6 signalling (classic and trans-signalling) with potential functions during SARS-CoV-2 infection is provided (**Supplementary Figure S3**).

## Discussion

This study was designed to investigate markers with capacity to predict severity of COVID-19 infection focusing on IL-6 signalling. We demonstrate that IL-6 signalling-related biomarkers (IL-6, sIL-6R, sgp130, IL-6 trans-signalling estimated by the B/T complex ratio and the FME) can predict the evolution of the disease in COVID-19 patients. Moreover, models combining IL-6 signalling variables have better diagnostic and prognostic power than individual analyses of each component. We provide new information on the incompletely understood role of IL-6 signalling in COVID-19 patients, which might be useful not only as biomarkers of severity but also in designing new treatment strategies. To the best of our knowledge, this is the first study analysing the association of the potential IL-6 trans-signalling with the risk of future outcome in SARS-CoV-2 infection.

Our cohort was initially classified into moderate and severe patients, distinguishing two groups in the latter category, severe survivors and severe non-survivors. Similar to recent studies, our data showed that IL-6 was significantly increased in severe groups (35). However, the levels of IL-6 were lower compared to those observed in sepsis (983 pg/mL), in non-COVID ARDS (460 pg/mL), and in CAR-T CRS (3110 pg/mL) (36), suggesting that the systemic inflammation during COVID-19 is less robust (37–39). In this regard, some authors have considered that COVID-19 is different in terms of scale of sensitivity or response characteristics. Nevertheless, it is important to mention that in the interpretation of the serum IL-6 levels, an absolute concentration of this cytokine could be useless, given its interactions with soluble receptors and its impact on the different modes of signalling. Thus, understanding the balance of these factors is crucial for the accurate interpretation of the serum IL-6 measurements. Since IL-6, sIL-6R, and sgp130 form the active binary and inactive ternary IL-6 complexes on a molar level, the impact of the potential IL-6 trans-signalling pathway cannot be evaluated with the levels of IL-6 and the soluble receptors individually. Therefore, we used the B/T complex ratio and the combined analyses of IL-6 and FME to estimate IL-6 trans-signalling (17, 19, 31, 33).

Most investigations regarding IL-6 in SARS-CoV-2 infection have demonstrated that IL-6 increases prior to ARDS in critical COVID-19 patients (35) and that elevated IL-6 levels were predictors of the need for mechanical ventilation (40). Our results are in line with those studies in the context of severity of the disease. We tested the role of IL-6 signalling components as risk factors for poor outcome (ICU requirement or death). Statistical analyses showed that all of them had high ability to predict severity, both as independent variables and as co-variates. Notably, the scores combining IL-6 + sIL-6R + sgp130 exhibited an excellent classification performance for severity in survivor patients, improving the AUROC, sensitivity, specificity, and accuracy of the individual analyses. The data were supported by the highest length of hospital stay in patients with values of the three variables above cut-offs. Regarding IL-6 trans-signalling, the combination of IL-6 + FME improved the capacity to predict severity compared to IL-6 alone. Interestingly, sIL-6R, sgp130, and B/T complex ratio, but not IL-6, positively correlated with the levels of ferritin only in the severe group, supporting evidence that IL-6 trans-signalling could be associated with markers of severity and tissue damage. These findings reinforce the idea that IL-6 trans-signalling could play a role in the severity of COVID-19 and IL-6 trans-signalling related variables could be biomarkers in the progression of the disease rather than the levels of IL-6. Altogether, the data demonstrate the importance of analysing all IL-6 signalling components to obtain a robust predictive model for severity.

In severe survivors, most of the patients had high levels of sIL-6R as it has been reported recently (28), which may be explained by the release from activated T cells, a major source of sIL-6R (41). Indeed, it has been reported that severe COVID-19 patients show a T cell hyperactivation. The data support the idea that sIL-6R may be a helpful biomarker. However, patients with high levels of IL-6 did not have increased levels of sIL-6R in line with the observation by Koutsakos et al. (28). Here, it might argue that in some local settings these systemic changed ratios between IL-6, sIL-6R, and sgp130 might have consequences on local classic- and trans-signalling; which would determinate for the prognosis. Notably, in this group we found high levels of sgp130. The most probable explanation, in the context of the hyperinflammatory state, is that increased sIL-6R levels in combination with sgp130 would act as a buffer neutralising IL-6 trans-signalling, and this mechanism would account to avoid the fatal outcome of the disease (death).

Surprisingly, when we focused on non-survivors (exitus),the most relevant results were that sIL-6R, sgp130, and B/T complex ratio decreased significantly compared to severe patients, while IL-6 and FME increased. Remarkably, non-survivors exhibited a significant lymphopenia with increased levels of creatinine. The positive correlation between the levels of neutrophils and leukocytes with the time lapse to death suggests the existence of immune deficiency against viral infection in COVID-19, as it has been previously reported (42). Importantly, those variables (IL-6 signalling components and biochemical data) were independent predictors for mortality, and the combination of IL-6 + FME + lymphopenia + creatinine had the highest classification performance, significantly improving the predictive capacity of the independent risk factors. B/T complex ratio + lymphopenia + creatinine showed the highest OR to death. Supporting the role of the IL-6 variables in the fatal outcome, patients with increased levels of IL-6 and decreased levels of sIL-6R and sgp130 presented the lowest time to death. Although IL-6 signalling components served as independent predictors of death within 30 days, the models including B/T complex ratio and IL-6 + FME with lymphopenia + creatinine showed the most significant divergences in the overall survival outcome.

It is important to note that high values of the B/T complex ratio indicates that IL-6:sIL-6R binary complexes are not totally neutralized. Intriguingly, in the exitus group B/T complex ratio levels decreased in non-survivors compared to severe survivors, implying that the severe event (death) in this group was related with values below cut-off, in clear contrast with the severe survivors. Nevertheless, the exitus group presented the highest levels of IL-6. We can speculate that in the context of severe patients with marked lymphopenia and high levels of IL-6, values of the B/T complex ratio below cut-off or values of FME above cut-off, are useful predictors of a fatal outcome, probably reflecting a deep dysregulation of the protective immunity, that could be responsible for the death in these patients (43, 44). Interestingly, we observed that circulating levels of ferritin, which is used as a guide for anti-inflammatory treatments in patients with severe COVID-19 (45–47), decreased significantly in exitus compared with severe survivors, suggesting that the amplification of the inflammatory response has already occurred. Altogether, we propose that in severe patients with signs of immunosuppression milieu (48), as it has been reported in severe sepsis (49) and speculated in severe COVID-19 with high mortality (44), a low B/T complex ratio would indicate a failure in the regulation of the immune protective system with higher sensitivity to death. The increased levels of IL-6 would be useful to identify patients at risk of death in this group. Moreover, we could speculate that the use of immunomodulators and/or IL-6 blockers in these patients would not provide benefits, in clear contrast with the severe group in which the inflammatory response would be amplified together with high sIL6-R levels. Further investigations are needed in order to clarify the potential role of trans-signalling in those conditions.

Among the main limitations of this study was the analysis of disease severity, which might differ from classifications used in other studies. Nevertheless, our study’s pragmatic design is in line with other studies (26–28), and it could better reflect the characteristics of our cohort. In addition, we could not stratify patients based on the treatment received. It is important to note that about 80% of the patients in this study were recruited between March and April 2020, when the pandemic was in the first wave and knowledge regarding patient’s stratification and treatments were very limited. This circumstance minimizes chances that our results were influenced by treatments.

In conclusion, our study suggests that the screening of IL-6 signalling components at the hospital admission can identify patients at risk of severe COVID-19. Nevertheless, the levels of IL-6, sIL-6R, and sgp130 markers might be evaluated in the clinical context. Altogether, we identify novel biomarkers that may constitute a helpful tool for the early identification, stratification of patients at hospital entry, and prediction of the disease progression in terms of severity and/or death, with clear implications in treatment and clinical decision-making.

## Data Availability Statement

The raw data supporting the conclusions of this article will be made available by the authors, without undue reservation.

## Ethics Statement

The studies involving human participants were reviewed and approved by Biomedical Research Ethics Committee of Andalucía code: COVIDIL6, and Andalucía Biobank code: S2000228. The patients/participants provided their written informed consent to participate in this study.

## Author Contributions

MAR-H, JH-Q, FG, SR-J, and MB conceptualized and designed the study. MAR-H, DC, GL-R, MN-N, RC, RB, AF-L, MEC-S, AM-T, JH-Q, and FG conducted the investigations (recruiting patients or conducting laboratory tests). MAR-H, JP-F, and MB conducted formal data analysis. MAR-H, CG, SR-J, and MB wrote the original first draft of manuscript. All authors reviewed the manuscript and gave significant input. MAR-H and MB had access to and verified all underlying data. The final version of this paper was reviewed and approved by all authors.

## Funding

The research work was supported by the Spanish Institute of Health Carlos III (COV-20/00792) and by the European Commission - NextgenerationEU (Regulation EU 2020/2094), through CSIC's Global Health Platform (PTI Salud Global). MAR-H acknowledges support from the Spanish Institute of Health Carlos III and the European Commission - NextgenerationEU (Regulation EU 2020/2094), through CSIC's Global Health Platform (PTI Salud Global). DC is supported by a predoctoral iPFIS (IFI 19/00048) funded by Spanish Institute of Health Carlos III. MN-N is supported by the Rio Hortega contract (CM20/00074).

## Conflict of Interest

The authors declare that the research was conducted in the absence of any commercial or financial relationships that could be construed as a potential conflict of interest.

## Publisher’s Note

All claims expressed in this article are solely those of the authors and do not necessarily represent those of their affiliated organizations, or those of the publisher, the editors and the reviewers. Any product that may be evaluated in this article, or claim that may be made by its manufacturer, is not guaranteed or endorsed by the publisher.

## Abbreviations

ARDS, acute; Respiratory Distress Syndrome; AUROC, area under receiver operating characteristic; B/T complex ratio, Binary/Ternary complex ratio; CAR-T, Chimeric Antigen Receptor-modified T cells; CI, Confidence Interval; COVID-19, Coronavirus Disease-19; CPR, C Reactive Protein; CRS, Cytokine Release Storm; FME, Fold Molar Excess of sgp130 over sIL-6R; HR, Hazard Ratio; IL-6, Interleukin 6; NPV, Negative Predictive Value; PPV, Positive Predictive Value; OR, Odds Ratio; sIL6-R, soluble Interleukin 6 Receptor; sgp130, soluble gp130; SARS-CoV-2, Severe acute; Respiratory Syndrome-Related Coronavirus 2.
