# Supplementary figures and images for: Identification of IL-6 Signalling Components as Predictors of Severity and Outcome in COVID-19
Source: Front Immunol. 2022 May 13;13:891456. doi: 10.3389/fimmu.2022.891456 (PMC9137400; doi:10.3389/fimmu.2022.891456)

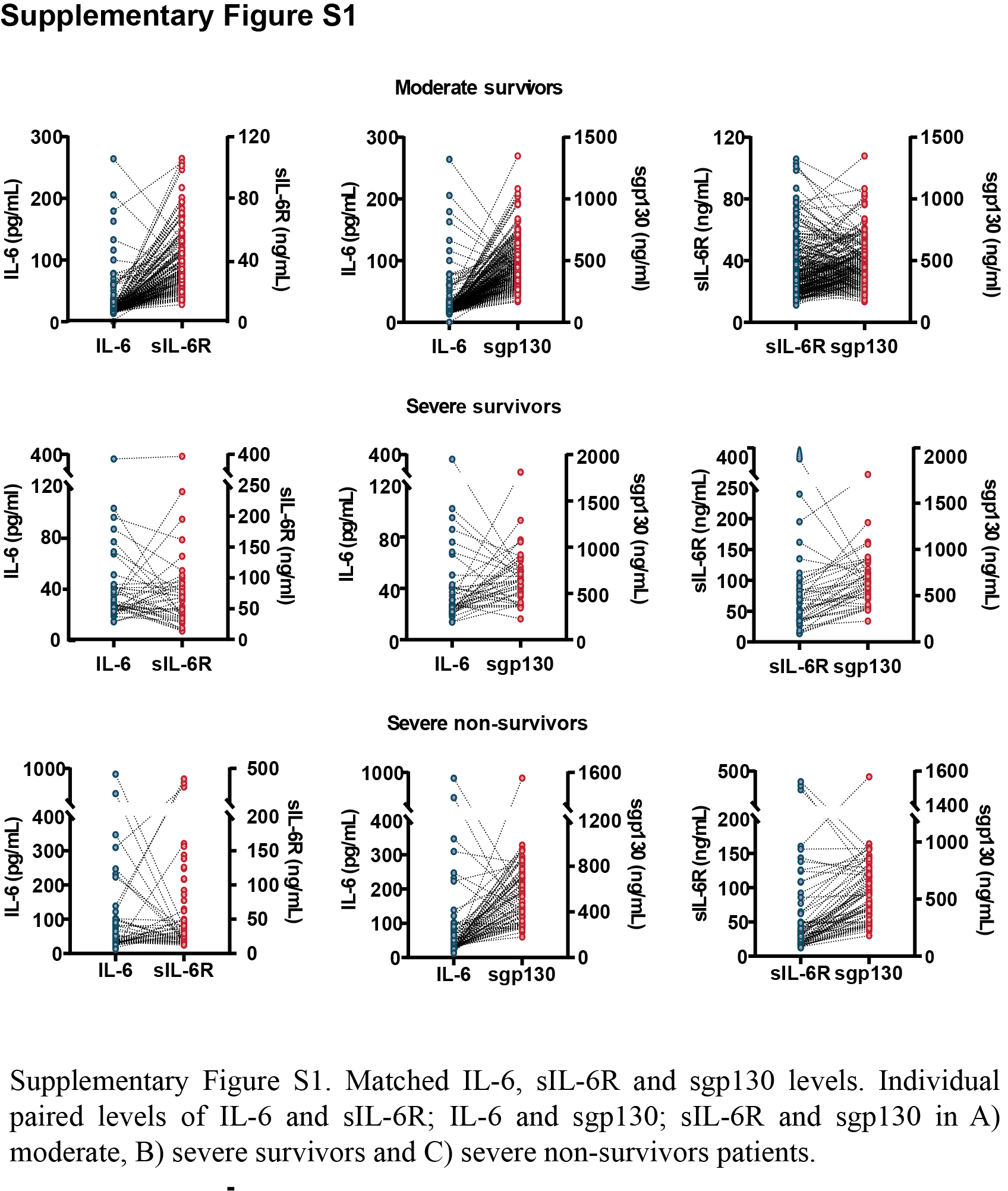

Supplement: Supplementary file 1 [file Image_1.tif]

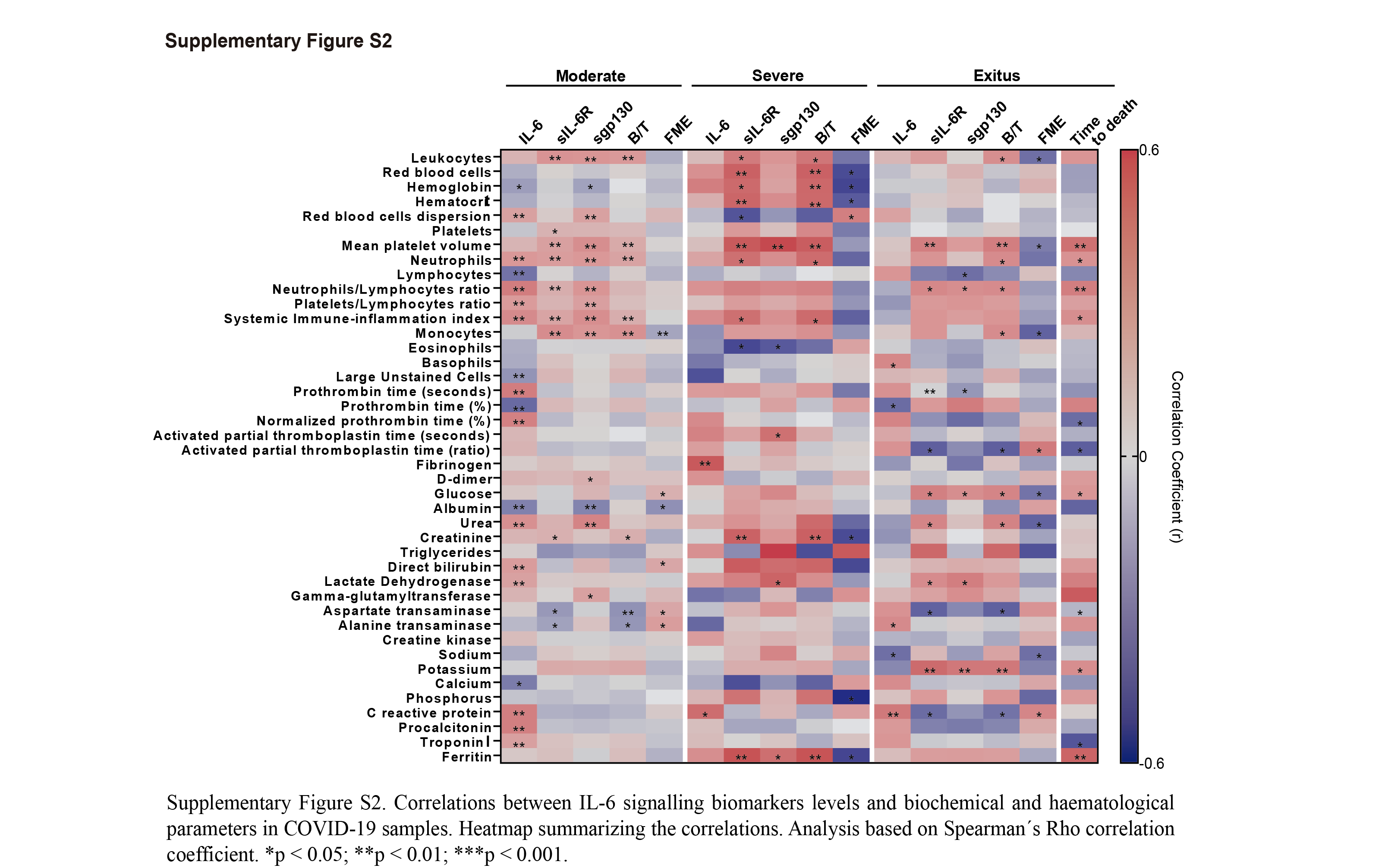

Supplement: Supplementary file 2 [file Image_2.tif]

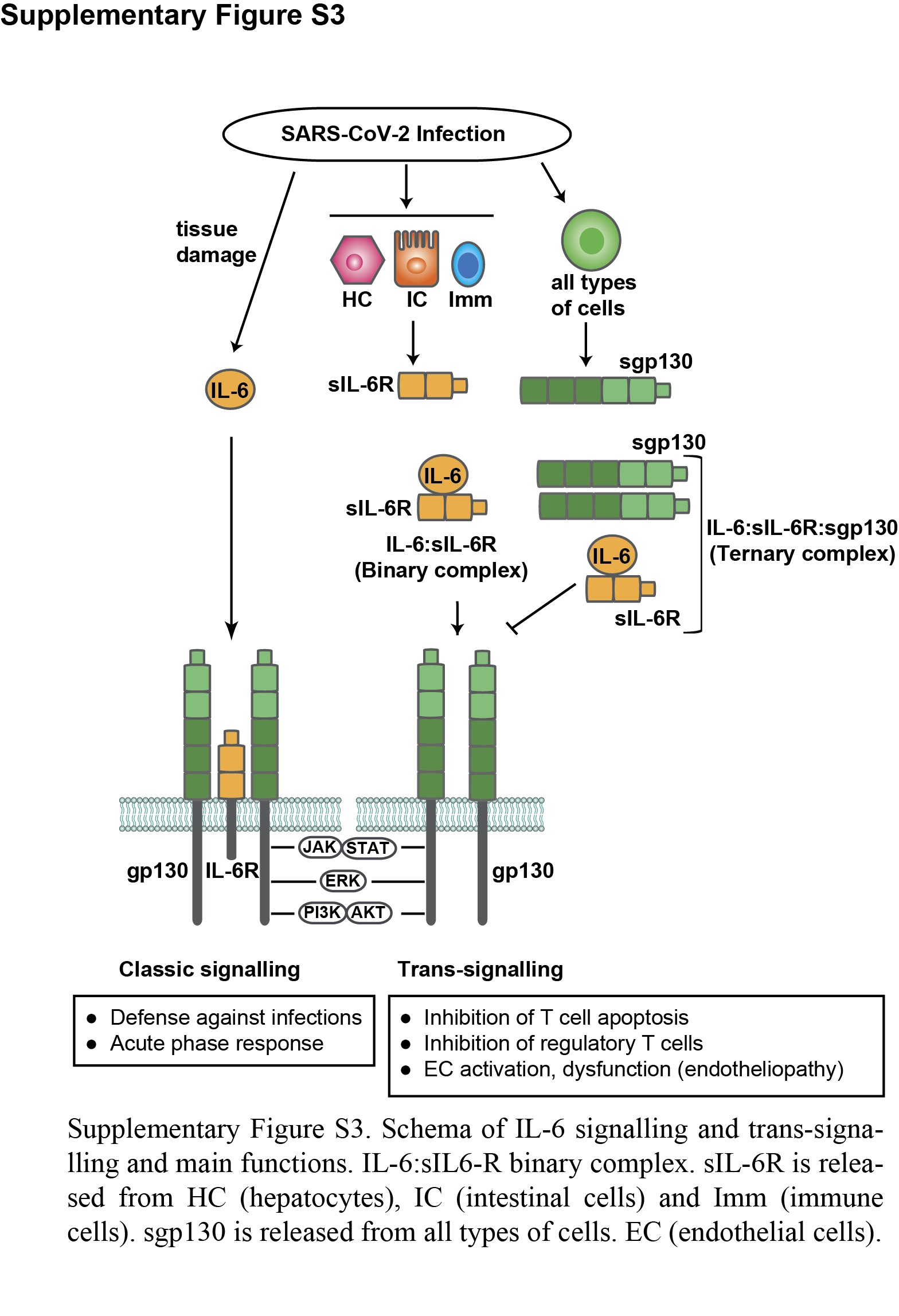

Supplement: Supplementary file 3 [file Image_3.tif]
