# Supplementary material for: Identification of IL-6 Signalling Components as Predictors of Severity and Outcome in COVID-19
Source: Front Immunol. 2022 May 13;13:891456. doi: 10.3389/fimmu.2022.891456 (PMC9137400; doi:10.3389/fimmu.2022.891456)
Supplement: Supplementary file 4 [file Table_1.docx]

| **Supplementary Table S1.** Demographic and clinical characteristics of the cohort at the hospitalization admission. Continuous variables are presented as median (IQR) and categorical variables are presented in percentages in the whole cohort and each subgroup. IQR, Interquartile range; NA, not applicable. | | | | |
| --- | --- | --- | --- | --- |
|  |  |  |  |  |
|  | **Overall cohort** | **Moderate** | **ICU** | **Exitus**  **(severe non-survivors)** |
|  |  | **(ward)** | **(severe survivors)** |  |
|  | (n=366) | (n = 257 [70.20 %]) | (n = 40 [10.93 %]) | (n = 69 [18.85 %]) |
| Age (y), median (IQR) | 68.91 (56 - 80) | 67.14 (54.09 - 78.25) | 62.46 (49.08 – 70.00)*** | 78 (69.04 – 87.22)***, ^###^ |
| Gender, number (%) |  |  |  |  |
| male | 192 (52.46) | 126 (49.03) | 24 (60) | 42 (60.87)*^,###^ |
| female | 174 (47.54) | 131 (50.97) | 16 (40) | 27 (39.13) |
| **Previous comorbidities** |  |  |  |  |
| Hypertension | 177 (48.36) | 115 (44.75) | 13 (32.5) | 49 (71)** |
| Diabetes mellitus | 94 (25.68) | 61 (23.74) | 10 (25) | 25 (36) |
| Dyslipidemia | 96 (26.23) | 61 (23.74) | 10 (25) | 25 (36.2)* |
| Obesity | 55 (15.03) | 33 (12.84) | 9 (22.5) | 13 (18.8) |
| Neoplasia | 26 (7.10) | 22 (8.56) | 1 (2.5) | 3 (4) |
| Chronic pulmonary disease | 101 (27.60) | 59 (22.96) | 15 (37.5)* | 27 (39.1)* |
| Chronic hepatic disease | 23 (6.28) | 16 (6.23) | 2 (5) | 5 (7.2) |
| Chronic heart disease | 77 (21.04) | 43 (16.73) | 7 (17.5) | 27 (39)** |
| Chronic renal disease | 30 (8.2) | 16 (53.3) | 0 | 14 (20.3)***^, ###^ |
| Stroke | 31 (8.47) | 20 (7.78) | 3 (7.5) | 4 (5.8) |
| **Mechanical ventilation** | 44 (12) | 0 (0) | 24 (60)*** | 20 (29)***,^###^ |
| **Radiological evidence of pneumonia** | 258 (76.3) | 175 (71.6) | 34 (97.2)** | 49 (83.1)**^,#^ |
| **Exitus, number ( %)** | 69 (18.85) | 0 (0) | 0 (0) | 69 (100)***^, ###^ |
| **COVID-19 treatment** |  |  |  |  |
| Lopinavir/Ritonavir | 181 (49.45) | 154 (59.92) | 11 (27.50)*** | 16 (23.2)*** |
| Hidroxicloroquina | 217 (56.8) | 178 (69.3) | 7 (17.50)*** | 23 (33.3)**^,###^ |
| Azitromicina | 191 (52.19) | 159 (61.87) | 12 (30.00)*** | 20 (29)^###^ |
| Ceftriaxona | 63 (17.21) | 32 (12.45) | 15 (37.50)*** | 16 (23.2) |
| Linezolid | 21 (5.74) | 3 (1.17) | 9 (22.50)*** | 9 (13)**^.##^ |
| Meropenem | 22 (6.01) | 3 (1.17) | 12 (30.00)*** | 7 (10.1)**^.##^ |
| Tociluzimab | 21 (5.74) | 8 (3.11) | 3 (7.50) | 10 (14.5)*** |
| Anakinra | 6 (1.46) | 1 (0.39) | 1 (2.50) | 4 (5.8)^##^ |
| Interferon-β | 8 (2.19) | 3 (1.17) | 4 (10.00)*** | 1 (1.4)* |
| Levofloxacino | 10 (2.73) | 1 (0.39) | 4 (10.00)*** | 5 (7.2)^#^ |
| Corticosteroids | 91 (24.86) | 48 (18.68) | 22 (55)*** | 21 (30.44)*^.#^ |
| **Symptoms** |  |  |  |  |
| Oxygen saturation ≤93 | 186 (50.82) | 86 (33.46) | 36 (90)*** | 63 (91.67)*** |
| Diarrhea | 88 (24.00) | 68 (26.5) | 8 (20.0) | 12 (17.4) |
| Dyspnea | 183 (50.00) | 115 (44.7) | 29 (72.5)*** | 39 (56.5)* |
| Fever | 249 (68.00) | 190 (73.9) | 27 (67.5) | 32 (46.4)***^, #^ |
| Headache | 67 (18.3) | 53 (20.6) | 7 (17.5) | 7 (10.1) |
| Myalgia | 83 (22.7) | 63 (24.5) | 11 (27.5) | 9 (13.0)* |
| Cough | 200 (54.6) | 146 (56.8) | 21 (52.5) | 33 (47.8) |
| Anosmia | 47 (12.8) | 43 (16.7) | 1 (2.5)* | 3 (4.3)* |
| Dysgeusia | 56 (15.3) | 52 (20.2) | 1 (2.5)*** | 3 (4.3)*** |
| Asthenia | 108 (29.5) | 87 (33.9) | 8 (20.0) | 13 (18.8)* |
| *p < 0.05, **p < 0.01; ***p < 0.001 *vs* Moderate group; ^#^p < 0.05, ^##^p < 0.01; ^###^p < 0.001 *vs* Severe survivor group. | | | | |
