# Supplementary material for: Identification of IL-6 Signalling Components as Predictors of Severity and Outcome in COVID-19
Source: Front Immunol. 2022 May 13;13:891456. doi: 10.3389/fimmu.2022.891456 (PMC9137400; doi:10.3389/fimmu.2022.891456)
Supplement: Supplementary file 5 [file Table_2.docx]

| **Supplementary TABLE S2.** Laboratory findings in COVID-19 cohort. Continuous variables are presented as median (IQR) and categorical variables are presented in percentages in the whole cohort and each subgroup. Results corresponded to the first available blood sample after hospital admission (median:1 day; IQR: 1-4). IQR, Interquartile range; NA, not applicable. | | | | | | | | |
| --- | --- | --- | --- | --- | --- | --- | --- | --- |
|  | **Overall cohort** | | **Moderate (ward)** | | **ICU (severe survivors)** | | **Exitus (severe non-survivors)** | |
|  |  |  |  |  |  |  |  |  |
|  | n | median (IQR) | n | median (IQR) | **n** | **median (IQR)** | **n** | **median (IQR)** |
| Leukocytes (number, x 10^3/µL) | 359 | 6.45 (4.85 - 8.98) | 253 | 5.76 (4.46 - 7.42) | 40 | 9.12 (7.105 -11.88)*** | 66 | 9.65 (6.8525 -14.115)*** |
| Red blood cells (number, x 10^6/µL) | 360 | 4.58 (4.1 - 4.97) | 254 | 4.65 (4.27 - 5) | 40 | 4.49 (3.85 -5.01) | 66 | 4.12 (3.705 -4.66)***^, #^ |
| Hemoglobin (g/dL) | 347 | 13.5 (12 - 14.6) | 246 | 13.6 (12.5 - 14.7) | 37 | 13.6 (11.8 -14.65) | 64 | 12.2 (10.725 -14.15)***^, #^ |
| Hematocrit (%) | 361 | 38.9 (35.3 - 42.5) | 255 | 39.5 (36.5 - 42.5) | 40 | 39.35 (35.225 -43.65) | 66 | 36.25 (32.325 -41.375)***^, #^ |
| Mean corpuscular hemoglobin (pg) | 361 | 29.8 (28.5 - 30.9) | 255 | 29.7 (28.5 - 30.9) | 40 | 87.18 (81.8 -92.55) | 66 | 88.49 (82.32 -94.66)*** |
| Mean corpuscular hemoglobin concentration (g/dL) | 360 | 34.2 (33.3 - 35.1) | 254 | 34.4 (33.5 - 35.1) | 40 | 29.7 (28.1 -30.8) | 66 | 30.05 (28.3 -30.975)*** |
| Red blood cells dispersion (%) | 361 | 13.8 (13.1 - 14.8) | 255 | 13.6 (13 - 14.7) | 40 | 13.55 (13.025 -14.275) | 66 | 14.5 (13.8 -15.725)***^, #^ |
| Platelets (number, x 10^3/µL) | 359 | 224 (169 - 299) | 253 | 218 (173 - 290) | 40 | 249 (199.25 -320)* | 66 | 221 (150.5 -298)^#^ |
| Mean platelet volume (fL) | 360 | 8.8 (7.9 - 10) | 255 | 8.4 (7.7 - 9.5) | 40 | 10.3 (8.625 -11.15) | 65 | 9.7 (8.4 -10.65)* |
| Neutrophils (number, x 10^3/µL) | 361 | 4.38 (3.07 - 7.38) | 255 | 3.75 (2.68 - 5.26) | 40 | 7.90 (5.08 -10.38)*** | 66 | 7.90 (5.56 -12.49)*** |
| Lymphocytes (number, x 10^3/µL) | 361 | 1.1 (0.72 - 1.54) | 255 | 1.22 (0.86 - 1.63) | 40 | 0.94 (0.655 -1.195)*** | 66 | 0.68 (0.405 -1.125)***^, #^ |
| Neutrophils/Lymphocytes (ratio) | 361 | 4.03 (2.34 - 8.42) | 255 | 3.02 (2.02 - 5.19) | 40 | 8.28 (5.33 -12.60)*** | 66 | 11.68 (6.43 -25.31)***^, #^ |
| Platelets/Lymphocytes (ratio) | 359 | 207.91 (143.61 - 304.85) | 253 | 186.84 (134.86 - 261.94) | 40 | 297.93 (204.17 -388.02)*** | 66 | 291.25 (183.98 -600.67)*** |
| Systemic Immune-inflammation index (ratio) | 359 | 906.38 (485.95 - 2075.82) | 253 | 678.28 (431.91 - 1224.49) | 40 | 2491.71 (1180.39 -3597.82)*** | 66 | 2260.66 (1109.08 -6514.6)*** |
| Monocytes (number, x 10^3/µL) | 361 | 0.4 (0.27 - 0.55) | 255 | 0.4 (0.29 - 0.52) | 40 | 0.495 (0.2525 -0.665) | 66 | 0.375 (0.2475 -0.6025) |
| Eosinophils (number, x 10^3/µL) | 359 | 0.02 (0 - 0.07) | 254 | 0.03 (0.01 - 0.09) | 39 | 0.01 (0 -0.04) | 66 | 0.01 (0 -0.0325)*** |
| Basophils (number, x 10^3/µL) | 360 | 0.02 (0.01 - 0.03) | 254 | 0.02 (0.01 - 0.03) | 40 | 0.02 (0.01 -0.04) | 66 | 0.01 (0.01 -0.03) |
| Large Unstained Cells (number, x 10^3/µL) | 300 | 0.1 (0.07 - 0.14) | 234 | 0.1 (0.07 - 0.13) | 18 | 0.095 (0.0675 -0.1825) | 48 | 0.1 (0.07 -0.14) |
| Prothrombin time (seconds) | 244 | 10.7 (10.2 - 11.7) | 164 | 10.5 (10.1 - 11) | 30 | 12.25 (10.975 -12.975)*** | 50 | 11.2 (10.6 -12.625)*** |
| Prothrombin time (%) | 243 | 98 (89 - 104) | 162 | 100 (93 - 105) | 31 | 90 (78 -102)** | 50 | 91 (77.75 -102.25)** |
| Normalized prothrombin time (%) | 238 | 1 (0.95 - 1.07) | 157 | 0.99 (0.95 - 1.04) | 31 | 1.05 (0.99 -1.17)** | 50 | 1.05 (0.98 -1.17)*** |
| Activated partial thromboplastin time (seconds) | 237 | 24.7 (22.3 - 28.3) | 158 | 24 (22 - 26.2) | 31 | 28.6 (21.3 -32.7)** | 48 | 27.3 (24.05 -31.25)*** |
| Activated partial thromboplastin time (ratio) | 238 | 0.93 (0.85 - 1.02) | 160 | 0.92 (0.85 - 1) | 30 | 0.925 (0.7875 -1.0325) | 48 | 0.99 (0.8775 -1.18)** |
| Fibrinogen (mg/dL) | 241 | 350 (350 - 400) | 161 | 350 (350 - 400) | 30 | 594.5 (430.5 -657.25)*** | 50 | 400 (397.25 -489)***^, ##^ |
| D-dimer (ng/mL) | 324 | 810 (435 - 1710) | 229 | 690 (380 - 1280) | 35 | 1510 (660 -2550)*** | 60 | 1420 (792.5 -3930)*** |
| Glucose (mg/dL) | 340 | 104 (85 - 141.5) | 238 | 93.5 (84 - 120) | 37 | 123 (94 -173)** | 65 | 138 (107 -197)*** |
| Albumin (g/dL) | 269 | 3.6 (3.3 - 4) | 187 | 3.7 (3.5 - 4.1) | 30 | 3.3 (2.975 -3.525)*** | 52 | 3.2 (2.7 -3.6)*** |
| Total proteins | 230 | 6.9 (6.4 - 7.3) | 152 | 7 (6.6 - 7.4) | 31 | 6.5 (6.2 -7.2)** | 47 | 6.6 (5.9 -7.3)** |
| Urea (mg/dL) | 161 | 49 (31 - 83) | 92 | 39 (27 - 65) | 22 | 48.5 (30 -73.25) | 47 | 84 (53 -118)*** |
| Creatinine (mg/dL) | 352 | 0.85 (0.68 - 1.105) | 246 | 0.835 (0.67 - 1) | 38 | 0.795 (0.65 -1.06) | 68 | 1.195 (0.805 -1.632)***^, ##^ |
| Cholesterol (mg/dL) | 145 | 142 (121 - 167) | 116 | 145.5 (123.5 - 169) | 11 | 130.94 (77.44 -184.44) | 18 | 129.5 (94.6 -164.94) |
| Triglycerides (mg/dL) | 128 | 124.5 (91.5 - 181.5) | 101 | 115 (90 - 175) | 6 | 222.5 (84.3 -340.25) | 21 | 142 (93.5 -201.5) |
| Total bilirubin (mg/dL) | 327 | 0.61 (0.47 - 0.9) | 230 | 0.63 (0.47 - 0.89) | 36 | 0.585 (0.4025 -0.875) | 61 | 0.6 (0.4 -1) |
| Lactate Dehydrogenase (U/L) | 333 | 291 (230 - 387) | 238 | 260 (218 - 324) | 34 | 469 (303 -563.75)*** | 61 | 442 (308 -535)*** |
| Gamma-glutamyltransferase (U/L) | 218 | 40 (24 - 88) | 150 | 34 (24 - 75) | 27 | 65 (37 -107) | 41 | 42 (23 -98) |
| Aspartate transaminase (U/L) | 334 | 33 (24 - 46) | 238 | 32 (24 - 42) | 35 | 37 (30 -57)* | 61 | 34 (22.5 -49.5) |
| Alanine transaminase (U/L) | 340 | 27 (18 - 42) | 240 | 27 (18 - 39.5) | 37 | 40 (26 -60.5)*** | 63 | 23 (18 -35)^###^ |
| Creatine kinase (U/L) | 223 | 78 (39 - 154) | 147 | 76 (43 - 138) | 29 | 91 (32 -241) | 47 | 94 (47 -263) |
| Sodium (mEq/L) | 303 | 138 (136 - 140) | 207 | 137 (135 - 139) | 37 | 140 (138 -143)** | 59 | 139 (136 -143)** |
| Potassium (mEq/L) | 296 | 4 (3.6 - 4.4) | 201 | 4 (3.6 - 4.4) | 36 | 3.9 (3.6 -4.2)** | 59 | 4.2 (3.6 -4.7) |
| Calcium (mg/dL) | 163 | 8.9 (8.4 - 9.4) | 110 | 9.1 (8.7 - 9.4) | 23 | 8.5 (8.1 -9)** | 30 | 8.4 (8.025 -8.925)*** |
| Phosphorus (mg/dL) | 140 | 2.9 (2.4 - 3.4) | 96 | 2.8 (2.4 - 3.3) | 19 | 3 (2.3 -3.6) | 25 | 2.9 (2.1 -3.45) |
| C reactive protein (mg/L) | 342 | 46.85 (14.5 - 115.9) | 239 | 30.7 (10 - 69.9) | 38 | 99 (37.4 -148.625)*** | 65 | 135.4 (68.75 -213.5)*** |
| Procalcitonin (ng/mL) | 222 | 0.08 (0.05 - 0.19) | 146 | 0.06 (0.04 - 0.1) | 30 | 0.11 (0.0575 -0.4425)** | 46 | 0.22 (0.1175 -0.6125)*** |
| Troponin I (ng/mL) | 195 | 7.4 (5 - 20.6) | 134 | 5.3 (4 - 9.8) | 16 | 9.7 (6.2 -34.475)** | 45 | 30.9 (13.3 -56.75)***^, ##^ |
| Ferritin (ng/mL) | 309 | 420.3 (197.8 - 722.1) | 225 | 332 (178.7 - 576.9) | 28 | 1079.8 (636.125 -1802.275)*** | 56 | 709.15 (357.9 -1355.325)***^, #^ |
| *p < 0.05, **p < 0.01; ***p < 0.001 *vs* Moderate group; ^#^p < 0.05, ^##^p < 0.01; ^###^p < 0.001 *vs* Severe survivor group. | | | | | | | | |
