# Supplementary material for: Identification of IL-6 Signalling Components as Predictors of Severity and Outcome in COVID-19
Source: Front Immunol. 2022 May 13;13:891456. doi: 10.3389/fimmu.2022.891456 (PMC9137400; doi:10.3389/fimmu.2022.891456)
Supplement: Supplementary file 6 [file Table_3.docx]

| **Supplementary TABLE S3-A. Adjusted multivariate logistic regression analyses of IL-6 signalling biomarkers as predictors of COVID-19 severity (without death).** | | | | |
| --- | --- | --- | --- | --- |
|  | **Cut-off multivariate logistic** | | **Cut-off multivariate logistic** | |
| **Variable** | **O.R.** | **95% CI** | **O.R.** | **95% CI** |
| **IL-6** (≥ 22.75 pg/mL) | 16.86 | (6.18-45.96))*** | 16.90 | (6.79-42.10)*** |
| **sIL-6R** (≥ 34.52 ng/mL) | 5.10 | (1.87-13.95)*** | - | - |
| **sgp130** (≥ 556.50 ng/mL) | 5.08 | (1.99-12.99)*** | - | - |
| **B/T** (≥ 1.591) | - | - | - | - |
| **FME** (≤ 2.855) | - | - | 5.07 | (2.21-11.61)*** |
| **Age** (≥ 69) | 4.34 | (6.18-45.96)*** | 1.14 | (0.50-2.60) |
| **Gender** *(Man)* | 1.68 | (0.68-4.11) | 2.41 | (1.02-5.71) |
| Negative predictive value (NPV); Positive predictive value (PPV); Binary/ternary complex ratio (B/T); Fold molar excess of sgp130 over sIL-6R (FME); Odds ratio (O.R.). ****p* < 0.001. | | | | |
|  |  |  |  |  |
| **Supplementary TABLE S3-B. Adjusted multivariate logistic regression analyses of IL-6 signalling biomarkers as predictors of COVID-19 mortality.** | | | | |
|  | **Cut-off univariate logistic** | | **Cut-off univariate logistic** | |
| **Variable** | **O.R.** | **95% CI** | **O.R.** | **95% CI** |
| **IL-6** (≥ 27.40 pg/mL) | 3.35 | (1.36-9.89)* | - | - |
| **B/T** (≤ 1.561) | - | (2.11-17.33)** | 6.02 | (1.55-23.41)*** |
| **FME** (≥ 2.869) | 4.29 | (1.33-13.89)** | - | - |
| **Lymphocytes** (≤ 0.565 x 10^3/µL) | 6.55 | (1.43-29.98)*** | 5.73 | (1.43-22.99)* |
| **Creatinine** (≥ 1.15 U/L) | 7.41 | (2.08-26.46)** | 8.49 | (2.45-29.45)*** |
| **Age** (≥ 72) | 6.46 | (1.43-29.98)** | 7.61 | (2.42-23.90)*** |
| **Gender** *(Man)* | 1.05 | (0.32-3.40) | 0.94 | (0.31-2.90) |
| Negative predictive value (NPV); Positive predictive value (PPV); Binary/ternary complex ratio (B/T); Fold molar excess of sgp130 over sIL-6R (FME); Odds ratio (O.R.). **p* < 0.05; ***p* < 0.01; ****p* < 0.001. | | | | |
